# The FHJ debate: Dental health is so crucial that it should be in the medical school curriculum

**DOI:** 10.1016/j.fhj.2026.100556

**Published:** 2026-09-30

**Authors:** Jasmine Ho, Clarissa Hjalmarsson

**Affiliations:** a Oral and Maxillofacial Surgery, Northwick Park Hospital, London North West University Healthcare NHS Trust, London, United Kingdom; b Department of Oral and Maxillofacial Surgery, King’s College Hospital, London, United Kingdom

**Keywords:** Medical education, Medical school curriculum, Interprofessional education, Integrated care, Oral Health

## Abstract

This debate article evaluates whether dental health education should be more widely integrated into the undergraduate medical curriculum. The authors present evidence-informed arguments situated within an evolving landscape of healthcare delivery, professional expectations, and political system-level pressures. Central to both perspectives is the need to advocate for patients’ best interests, along with the practicalities of implementing an integrated, cross-disciplinary healthcare curriculum. The discussion highlights the educational, clinical, and policy implications of closer alignment between medicine and dentistry, and considers of how best to prepare future doctors for holistic patient care.

## Proposition

Jasmine Ho

Dental health, encompassing the soft and hard tissues of the oral cavity, is an integral component of overall human health. For clarity, and in keeping with terminology used in the literature, the terms *‘*dental health’ and *‘*oral health’ will be used interchangeably throughout this proposition. The oral cavity is a complex anatomical and physiological system intimately connected to overall health. Periodontal disease, a chronic inflammatory condition of the supporting structures of the teeth, is strongly associated with systemic diseases, including cardiovascular disease, diabetes mellitus, pregnancy-related complications, rheumatoid arthritis, and cognitive decline.^1^ Furthermore, oral lesions frequently serve as early clinical indicators of systemic conditions such as Crohn’s disease, coeliac disease, anaemia, HIV infection, and haematological malignancies.^2^ The rising global incidence of oral cancer further underscores the necessity for early recognition by all clinicians, not solely dental specialists. Importantly, many oral diseases are preventable through timely intervention and appropriate health-promotion strategies. Consequently, oral health has gained prominence within public health policy and chronic disease management frameworks.^3^, ^4^ The American Surgeon General’s report emphasised that ‘oral health is essential to the general health and well-being of Americans,’ a position echoed by the World Health Organization global oral health programme, which advocates for the integration of oral health into broader healthcare provision to improve patient outcomes.^3^, ^5^

Medical practitioners will routinely encounter oral manifestations of systemic disease; knowing how to diagnose, manage and refer patients appropriately is a fundamental part of medical training. The proposition that dental health should be included in the medical school curriculum, therefore, rests on a simple but often overlooked principle: the mouth is part of the body. But despite this biological reality, oral health remains largely absent from undergraduate medical education,^6^ a gap that is increasingly difficult to justify.

The persistent exclusion of dental health from medical curricula reflects longstanding structural and institutional barriers. Medical and dental faculties have historically been geographically and administratively separate, and differences in pedagogical models have further hindered interdisciplinary collaboration.^7^, ^8^ Even in specialities such as Oral and Maxillofacial Surgery (OMFS), which inherently integrate medical and dental skill sets and knowledge, exposure remains inconsistent across medical schools.^6^ From a regulatory standpoint, the General Medical Council (GMC) medical education standards require graduates to demonstrate competence in managing complexity and uncertainty,^9^ a mandate that should logically extend to dental presentations, which frequently involve overlapping medical and dental pathology. Medical graduates must also demonstrate an understanding of health promotion, illness prevention, and the structure of the UK healthcare system. This would include aspects of oral health and NHS dentistry as outlined by Public Health England’s ‘All Our Health’ strategy.^10^ Given the well-established oral–systemic connection, omitting oral health from medical education neglects a critical diagnostic and therapeutic domain.

From the UK perspective, patients are increasingly turning to GPs and emergency departments with oral complaints, largely due to severe challenges in accessing National Health Service (NHS) dental services.^11^ This clearly presents as a symptom of a fragmented healthcare system in much need of both structural and contractual reforms, which unfortunately goes beyond the context of this debate. Nevertheless, it is clear that such systematic pressures would consequently place the onus on the medical providers to ‘fill in’ these service gaps. Hence, medical professionals should be prepared to manage and refer dental presentations appropriately, particularly when they are the first or only point of contact. However, surveys and studies among medical students worldwide consistently indicate poor oral health knowledge and management skills, underscoring the urgent need to address this curriculum gap.^6^, ^12^, ^13^, ^14^ The consequences of this gap are evident in current practice. From personal observations during OMFS on-calls, referrals from primary and secondary care often misidentify the affected tooth, misjudge the urgency of the condition, or fail to refer appropriately to community- or hospital-based dental services. Seeking help from medical professionals who may lack the necessary training to manage oral conditions increases the risk of misdiagnosis, inappropriate antibiotic prescribing, and delayed treatment.^15^, ^16^ In this context, the absence of oral health training is not merely an educational oversight, it can potentially present as a patient safety issue.^16^ Medical professionals play a pivotal role in health promotion, including aspects of dental health. Without formal dental health training, they cannot effectively reinforce preventive behaviours such as smoking cessation, reducing dietary sugars, or improving oral hygiene practices, nor can they collaborate optimally with dental colleagues.^15^ This undermines the NHS’s commitment to integrated, patient-centred care.^17^ Importantly, these shortcomings reflect systemic educational deficits rather than individual clinician failings. Encouragingly, studies indicate that medical professionals recognise their limitations and express a strong desire for further oral-health training.^12^, ^16^ This demand aligns with a growing international movement to integrate oral-health teaching into medical curricula.^18^ Several countries have successfully demonstrated this, including USA, Canada, Australia, Poland, Norway, Malaysia, Thailand, Taiwan and the Middle East.^13^, ^18^, ^19^, ^20^, ^21^, ^22^, ^23^, ^24^, ^25^ These models demonstrate that integration into the medical curriculum is both feasible and beneficial when both medical and dental fraternities collaborate effectively. Such formalised integration of the oral health module would also strengthen interprofessional collaboration between medical and dental professionals, improve patient outcomes, and support public health initiatives.

## Summary

In conclusion, integrating dental health, specifically oral health, into the medical school curriculum is a clinical, regulatory, and ethical imperative. The oral cavity is inseparable from systemic health, and in the current state of the NHS dental access crisis, it demands that medical practitioners be prepared to manage oral presentations safely. International models demonstrate that integration is achievable in medical education, and the consequence of inaction can affect clinical practice. Continued exclusion of dental health from medical education will likely compromise patient care, undermine public health objectives, and fail to reflect the realities of modern clinical medicine. The evidence is clear: aspects of dental health are so crucial that they must be included in the medical school curriculum.

## Opposition

Clarissa Hjalmarsson

Dental health is crucial; it is therefore imperative that it is addressed nationally by public health agencies, and that patients with dental pathologies are diagnosed and treated by appropriately trained and qualified dental professionals. Current medical training requires mastery of a wider breadth of knowledge than ever before. After qualification, doctors, and the practices and hospitals in which they work, are under intense individual and systemic pressures. Shifting the responsibility for dental health onto medics will not solve the problems of an NHS dental system that fails to meet patients’ needs, and increases the risk of delay, misdiagnosis and mismanagement.

As the 2023 Adult Oral Health Survey identified, over a fifth of dentate adults had at least one tooth with ‘extensive, obvious’ decay, representing a 13% increase since 2009. Seven per cent experienced dental pain or problems, 10% demonstrated pulpal involvement, ulceration, fistulation or dental abscess, and 43% reported that their oral health negatively affected their daily life.^26^ The UK NHS dental system is not currently meeting the needs of its population. One third of patients (35%) attend less than two-yearly, or only when experiencing dental concerns, with 40% of those unable to find a dentist and 31% unable to afford the charges.^26^ The Chief Dental Officer for England, Jason Wong, acknowledged that provision of NHS dental treatment in England has decreased since the Covid-19 pandemic, disproportionately affecting the most deprived. While recent NHS dental reforms attempt to improve access to emergency appointments, amongst other initiatives, these changes were identified as being a first step towards the required ‘future, more fundamental reforms’.^27^

However, the problems facing UK dental care will not be solved by shifting this burden onto doctors. Medical students already face a broader curriculum than ever before, navigating increasingly complex multimorbid patients, a new era of personalised medicine, and an ever-expanding array of drug treatments. As Lujan and DiCarlo highlight, information overload in medical training risks creating students who know more but understand less; the authors emphasise the importance of prioritising essential scientific knowledge rather than further bloating the existing curriculum with additional content.^28^

After graduation, medical doctors already see a high volume of dental patients. Significant numbers of patients with isolated dental pathology present to A&E and their GPs, driven by poor availability of NHS dental services, a reluctance or inability to pay for even subsidised dental care, or a poor understanding of their conditions. This increases the burden on already strained medical systems. Existing interventions to reduce A&E overcrowding and waiting times attempt to triage dental patients away from doctors. For example, the NHS 111 service aims to send patients with dental pathologies directly to dentists – the clinicians with the tools and training to help them. Arming medics with enough knowledge to re-triage patients back to dentists offers little benefit to patients or clinicians, and risks wasting the time of all involved.

This isn’t simply a question of knowledge; most dental pathologies are best investigated and managed using dental equipment unavailable in health settings. For example, a patient with severe toothache will be best managed by a detailed clinical examination of the tooth, specialised investigations including pulp sensibility testing and periapical radiographs, followed by procedures such as extirpation, pulp dressing or extraction. It is infeasible in our current system to expect medics to undertake these procedures, or to assume this diagnostic liability with only cursory undergraduate training. When patients present to medics with dental pain, they are often prescribed antibiotics pending definitive treatment by a dentist. In the absence of a dental abscess or specific risk factors, this is clinically unnecessary and contributes to antibiotic resistance.^29^

Dental professionals are better placed to help. Each year, over one thousand UK graduates, and a similar number of overseas-qualified dentists, join the GDC register, the latter of which is set to increase with planned improvements in exam availability.^30^ With five years of dental training, or three to four years on shortened graduate courses, dentists are better equipped to investigate, diagnose and treat dental pathologies, or refer onwards to specialist teams. Furthermore, as minimally-invasive dental care becomes the gold standard in dentistry, continuity of care helps patients to avoid unnecessarily aggressive treatment that risks early pulp death or tooth loss. Even simple advice on maintaining good oral health is better delivered by appropriately trained clinicians, including dental therapists, hygienists, and dentists, supported by national public health initiatives, than by overworked and under-resourced medical doctors struggling to remember details from their undergraduate training during a busy night on call. Similarly to medicine, continuity of care with appropriate professionals results in better dental outcomes.

Unquestionably, there are some dental-related health conditions that medics must recognise, for example a spreading deep neck space infection (including Ludwig’s angina), fractures to the upper and lower jaws, and severe dental bleeds. These potentially life-threatening emergencies require immediate resuscitation and prompt referral to the appropriate specialists, which in most cases are dual-qualified oral and maxillofacial surgeons. However, these conditions are covered in current medical undergraduate teaching, and the postgraduate curricula of related specialties such as RCEM and MRCGP. Similarly, medical students and doctors are already taught how to elicit red flag symptoms, recognise oral lesions concerning for cancer, and make an appropriate two-week wait referral to their local cancer unit.

## Summary

Dental health is crucial: for this reason it is imperative that patients are given timely care by appropriately qualified professionals. The current NHS dental system is not meeting the needs of the UK population. However, shifting the burden and liability of dental triage onto medical doctors in addition to their current responsibilities is not the answer. Outside a small number of critical conditions already covered in the medical curriculum which require referral to OMFS or Head and Neck cancer services, using doctors to triage dental pathologies wastes patients’ and clinicians’ time for little gain. Systems such as 111 already exist to try and reduce inappropriate dental presentations to A&E and GPs, while public health initiatives offer evidence-based population-level approaches to improve oral health. At a patient level, it is in everyone’s interests that dental pathologies are investigated, diagnosed and managed by appropriately qualified and resourced dentists. It is the task of government to ensure that this remains a viable option.

## CRediT authorship contribution statement

**Jasmine Ho:** Writing – review & editing, Writing – original draft, Supervision, Conceptualization. **Clarissa Hjalmarsson:** Writing – review & editing, Writing – original draft, Conceptualization.

## Funding

Neither author has received any form of funding or payment for this article.

## Declaration of competing interest

The authors declare the following financial interests/personal relationships which may be considered as potential competing interests: Jasmine Ho is an associate editor for Future Healthcare Journal. Both authors declare that they have no known competing financial interests that could have appeared to influence the work reported in this paper.
